# Monogenic forms of common variable immunodeficiency and implications on target therapeutic approaches

**DOI:** 10.1097/ACI.0000000000000947

**Published:** 2023-09-28

**Authors:** Giulio Tessarin, Manuela Baronio, Vassilios Lougaris

**Affiliations:** Pediatrics Clinic and Institute for Molecular Medicine ‘A. Nocivelli’, Department of Clinical and Experimental Sciences, University of Brescia and ASST Spedali Civili of Brescia, Brescia, Italy

**Keywords:** activated phosphoinositide 3-kinase δ syndrome, common variable immunodeficiency, cytotoxic T lymphocyte antigen 4, lipopolysaccharide-responsive beige-like anchor protein, target treatment

## Abstract

**Purpose of review:**

Common variable immunodeficiency (CVID) is the most common symptomatic inborn error of immunity. The disorder is characterized by variable clinical and immunological manifestations, and, in a small minority of patients, a monogenic cause may be identified. In this review, we focalized on three different monogenic forms of CVID-like disease.

**Recent findings:**

Activated phosphoinositide 3-kinase delta syndrome (APDS) is a rare disorder characterized by hyperactivated class I phosphatidylinositol-3 kinase (PI3K) pathway. Affected patients present with respiratory infectious episodes, impaired viral clearance and lymphoproliferation. Recently, a direct PI3K inhibitor has been approved and it showed encouraging results both in controlling clinical and immunological manifestations of the disease. On the other hand, patients with defects in *CTLA-4* or *LRBA* gene present with life-threatening immune dysregulation, autoimmunity and lymphocytic infiltration of multiple organs. Abatacept, a soluble cytotoxic T lymphocyte antigen 4 (CTLA-4) fusion protein that acts as a costimulation modulator, has been widely implemented for affected patients with good results as bridge treatment.

**Summary:**

Understanding the biological basis of CVID is important not only for enriching our knowledge of the human immune system, but also for setting the basis for potential targeted treatments in this disorder.

## INTRODUCTION

Common variable immunodeficiency (CVID) is the most prevalent symptomatic human inborn error of immunity (IEI), with a prevalence of 1 : 10 000–1 : 50 000 in Caucasians [1]. The clinical phenotype of affected patients is highly heterogeneous, encompassing increased susceptibility to infectious episodes, autoimmune phenomena, polyclonal lymphoproliferation, granulomatous disease, and increased risk in developing malignancy [2^▪^]. Rather than a single entity, CVID is considered as an umbrella term embracing an heterogenous group of clinical and immunological phenotypes, that may be caused by various genetic and/or environmental factors [2^▪^]. In fact, and conversely from other IEIs which are characterized by a specific genetic defect in only 10–30% of patients with a CVID reach a definite genetic diagnosis, while for the remaining patients the disease is considered polygenic/multifactorial rather than one based on mendelian inheritance [3,4]. Therapeutical strategies for CVID affected patients are mainly symptomatic, requiring life-long immunoglobulin replacement treatment (IgRT) as a mainstay; in addition, antimicrobial courses may be frequently required when infectious episodes occur, as well as immunosuppression/modulation for the management of autoimmune phenomena or chronic lymphoproliferation [5^▪^]. However, and with the exception of IgRT which reduces infectious episodes’ rates and severity, the natural history of noninfectious CVID-related complication is hardly predictable and empirical treatment is often adopted. Nonetheless, besides unveiling more insights on the human immune system biology, understanding the molecular basis of CVID and CVID-like disorders enables the physicians to exploit the molecular defect with targeted treatments. In this review, we will focus on monogenic forms of CVID for which a target treatment is currently available or under investigation. 

**Box 1:**
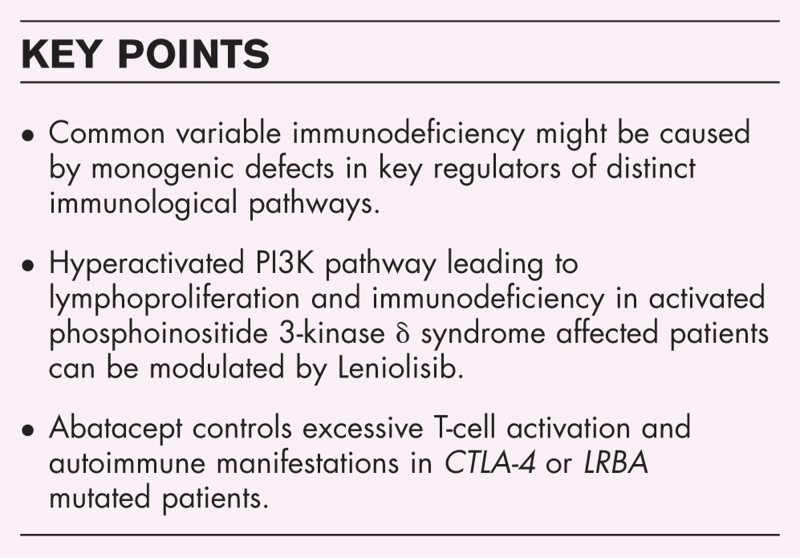
no caption available

## TEXT OF REVIEW

### Activated phosphoinositide 3-kinase delta syndrome

Class I phosphatidylinositol-3 kinases (PI3Ks) sense and transduce external signals which are fundamental for cell metabolism, differentiation, proliferation, and survival [6]. PI3Ks are formed by heterodimers comprising a regulatory (p85α, p55α, p50α, p85β, or p55γ) and a catalytic (class IA p110α, β, or δ; class IB p110γ) subunit; while p110α and β are ubiquitously expressed, p110δ and γ are primarily expressed in the immune system [6].

Activated phosphoinositide 3-kinase δ syndrome (APDS) is a recently described IEI characterized by recurrent sinopulmonary infectious episodes, bronchiectasis, impaired viral clearance, chronic lymphoproliferation, autoimmunity, and increased lymphoma risk [7]. The disease, inherited with an autosomal dominant manner, is caused by heterozygous gain-of-function mutations on the catalytic p110δ (coded by the *PIK3CD* gene, APDS-1, OMIM #615513) or by heterozygous loss-of-function mutations on the regulatory p85α (coded by the *PI3KR1* gene, APDS-2, OMIM #616005) subunit of the PI3K [8–11]. In both cases, a downstream hyperactivation of the PI3K pathway results in increased and sustained signaling on the mammalian/mechanistic target of rapamycin (mTOR) as well as c-Myc activation, which leads to metabolic reprogramming directed toward expansion and proliferation, ultimately resulting in an exhausted and senescent T-cell profile [12,13]. In addition, B cell signaling and maturation are also affected: impaired class-switch recombination and plasmablast maturation are observed, leading to poor humoral response, as well as loss of self-tolerance and survival of autoreactive immunoglobulin M (IgM)-secreting B cells, contributing to the development of autoimmune manifestations [14,15].

Since the first description of APDS in 2013, more than 250 patients have been described expanding the clinical and immunological features of the disease In fact, recurrent respiratory tract infectious episodes are the most common infectious manifestation in both APDS and CVID patients, as well as typical noninfectious complications such as autoimmune phenomena (i.e. enteropathy, autoimmune cytopenia, arthritis) or chronic benign lymphoproliferation (chronic adenopathies, hepatosplenomegaly, mucosal nodular lymphoid hyperplasia) [16^▪^,^17^,^18^]. On the contrary, additional nonimmunological features such as neurodevelopmental delay or neuropsychiatric disorders have been described in both APDS-1 and APDS-2 patients, manifestations, which are rather uncommon in CVID patients [18,19]. Peculiar immunological alterations that should raise suspicion for APDS are represented by raised IgM serum levels in a context of decreased IgG and IgA serum levels, progressive B cell lymphopenia with a relative expansion of transitional B cells and plasmablasts, decrease of naïve T cell subsets with relative expansion of central and effector memory T cells [16^▪^,^18^,^20^^▪▪^]. Nevertheless, taking in consideration the overlapping clinical phenotype of CVID and APDS patients, genetic screening including *PIK3CD* and *PIK3R1* should be offered to all genetically undefined CVID patients. The actual prevalence of APDS-causing mutations among CVID patients is difficult to estimate, as genetic analysis in CVID patients is not worldwide uniformed; however, data from large cohorts’ genetic studies revealed that pathogenic *PIK3CD* or *PIK3R1* mutations are found in approximately 1.4–7.1% of CVID patients [4,21,22]. Although displaying low genetic heterogeneity, APDS patients carrying the canonical E1021K mutation present highly variable clinical phenotype, thus suggesting a role for additional genetic/epigenetic or environmental factors in contributing to the disease manifestations [20^▪▪^]. The pathogenicity of novel unreported mutations must be confirmed by evaluating phosphorylation levels of AKT and/or S6 proteins in patients’ activated T or B lymphocytes [23].

Initially, treatment strategies for APDS patients resembled those adopted for CVID and were mainly symptomatic, including IgRT, antibiotic and/or antiviral treatment or prophylaxis, respiratory physiotherapy, and immunosuppressive drugs (e.g. corticosteroids, rituximab, azathioprine, mycophenolate, anti-TNFα monoclonal antibodies) [17,24,25]. However, and consistent with the hyperactivated mTOR pathway, a first attempt of tailored medicine was represented by the use of rapamycin: while highly effective on controlling chronic lymphoproliferation, it showed less robust results on cytopenias and colitis, even though with an adequate safety profile [17]. Hematopoietic stem cell transplantation (HSCT) represents the only curative approach for APDS; however, even though the results in terms of overall survival (OS) are satisfactory (86% 2-year OS), graft instability consequentially leading to graft failure requiring unplanned donor cell infusion represented a major limitation to successful HSCT [21,26^▪^,^27^^▪▪^]. Therefore, conservative treatment with the possibility of target therapy seems, at least for now, much more encouraging.

Recently, the results of a randomized, placebo-controlled, phase 3 trial of the PI3Kδ inhibitor Leniolisib have been published [28^▪▪^]. Leniolisib was administered to APDS patients aged 12 years or older at a dose of 70 mg twice daily over a period of 12 weeks; the drug was well tolerated with more drug-related adverse events in the placebo group rather than the Leniolisib one. Compared to placebo, Leniolisib effectively controlled chronic polyclonal lymphoproliferation, reducing lymph nodes as well as spleen size. Amelioration of immunological parameters such as increase of naïve B cell, decrease of serum IgM and improvement of autoimmune cytopenias also occurred. Finally, expanded transitional B cells and raised senescent CD8^+^ T cells, which are both thought to contribute to defective viral clearance, were both improved [28^▪▪^]. In March 2023, Leniolisib was approved in the United States by the Food and Drug Administration (FDA) for the treatment of APDS in adult or pediatric patients older than 12 years and is currently also under regulatory review in the European Union [29]. Besides Leniolisib, other PI3Kδ inhibitors have been investigated for APDS, with less promising results. An open-label trial of the inhaled agent Nemiralisib was completed in 5 APDS patients: even though Nemiralisib was well tolerated, the trial did not provide evidence regarding its efficacy in target engagement in the lung, as well as downstream effects modulating local lung or systemic blood inflammation, which could have been of benefit for APDS-affected patients [30].

### Cytotoxic T lymphocyte antigen 4 haploinsufficiency and lipopolysaccharide-responsive beige-like anchor protein deficiency

Cytotoxic T lymphocyte antigen 4 (CTLA-4) is a key T cell co-receptor which acts as a negative regulator in maintaining immune homeostasis by downregulating CD28:B7 ligands (CD80/CD86) interactions [31]. CTLA-4 plays its regulatory function both in a cell-extrinsic manner, where T-regulatory (T-reg) cells downregulate B7 by *trans*-endocytosis and degradation, and in a cell-intrinsic manner, by limiting B7 availability on the surface of T cells *via cis*-endocytosis [32^▪^,^33^]. *CTLA-4* haploinsufficiency causes a severe CVID-like monogenic IEI with predominantly immune dysregulatory features characterized by progressive B cell exhaustion and hypogammaglobulinemia, multiorgan autoimmunity (immune cytopenia, enteropathy, endocrinopathies), and chronic lymphoproliferation with lymphocytic infiltrates in several organs (brain, gut, liver, and lung) [34,35]. *CTLA-4* mutated patients express a highly heterogeneous clinical phenotype and incomplete disease penetrance: additional genetic or epigenetic, as well as environmental factors, are suspected to contribute to the clinical picture, even though this is still under investigation. Common infectious agents [such as Epstein−Barr virus (EBV) or cytomegalovirus (CMV)] were thought to act as a trigger; however, a recent study investigated the seroprevalence of EBV, CMV, Herpes simplex 1/2, Parvovirus B19 and Toxoplasma gondii among affected and unaffected *CTLA-4* mutated subjects, finding no differences [36^▪^].

Patients with biallelic loss-of-function mutations in the lipopolysaccharide-responsive beige-like anchor protein (*LRBA*) gene are clinical phenocopies of *CTLA-4* haploinsufficiency [37]. From a molecular point of view, LRBA prevents AP-1 driven CTLA-4 lysosomal degradation by recycling and cell surface transferring CTLA-4-containing vesicles; therefore, patients lacking wild-type LRBA expression display decreased CTLA-4 expression and altered T-reg function, thus causing immune dysregulation and autoimmunity [38]. Compared to *CTLA-4* mutated patients, *LRBA* deficiency affected patients tend to present a more severe clinical picture with earlier disease onset; the clinical picture comprises recurrent or invasive infectious episodes, enteropathy, autoimmune cytopenia, granulomatous disease, lymphoproliferation [39,40,41^▪^]. Immunological analysis revealed normal T cell count with reduced T-regs as well as B cells perturbations such as reduced switched-memory B cells and plasmablasts and expanded autoreactive CD21^lo^ B cells [39,40].

Even though both disorders affect the expression and/or function of the same immunological check-point inhibitor, *LRBA* deficient patients tend to present a more severe phenotype and a reduced OS – one possible explanation is given by a variable disease penetrance reported for *CTLA-4* mutated patients, whereas patients with *LRBA* deficiency normally present with complete penetrance and fully overt disease presentation [35,41^▪^]. Data from single-center or nationwide study revealed that the prevalence of pathogenic *CTLA-4* or *LRBA* mutations among subjects with a CVID/CVID-like diagnosis ranges between 1.7–20.8% and 0.9–7.2%, respectively [4,22]. Taking this into account, *CTLA-4* haploinsufficiency and *LRBA* deficiency represent two of the most common causes of monogenic CVID, therefore suggesting the vital importance of screening CVID patients for these two genes, both in terms of proper follow-up and due to the possibility of establishing a tailored treatment [4,42,43].

Besides IgRT for those patients presenting with hypogammaglobulinemia, additional treatment choices for patients with *CTLA-4* haploinsufficiency or *LRBA* deficiency are mainly directed towards autoimmune manifestations and/or organ lymphocytic infiltrates [39,44^▪▪^]. Conventional immunosuppressive drugs (systemic steroids, Mycophenolate Mophetile, Azathioprine) or T-cell modulators (Cyclosporine, Cyclophosphamide) were extensively used either alone or in combination to control autoimmune manifestations such as enteropathy or autoimmune cytopenias; in addition, the use of the mTOR inhibitor Sirolimus may enhance T-reg response [39,44^▪▪^]. B lymphocyte depleting therapy with anti-CD20 monoclonal antibodies showed good results in treating lymphoid infiltration, as B cells express CD80/CD86 and therefore T-cell activation can be limited [39,44^▪▪^].

Abatacept is a soluble fusion protein composed of the Fc IgG1 region and the CTLA-4 extracellular domain that selectively binds CD80/CD86, thus inhibiting the activation of CD28^+^ T cells [45,46]. Based on its biological function and with the rationale of providing exogenous CTLA-4 molecule, Abatacept has been implemented in several *CTLA-4* or *LRBA* mutated patients: dosing scheme for adult patients usually start with a loading dose of 500–1000 mg intravenously, followed then by 125–250 mg per week subcutaneously [44^▪▪^,^47^,^48^,49^▪^]. In pediatric patients with *CTLA-4/LRBA*-related disorders, Abatacept has be administered at a dose of 10–20 mg/kg every 2 weeks until complete remission, without major adverse events [49^▪^,^50^]. Even though Abatacept is reported to show high efficacy in controlling chronic enteropathy and lymphoproliferation, variable results with partial remission were seen in other immunedisregulatory features such as autoimmune cytopenia or neurological manifestations [44^▪▪^,^48^]. By contrast, in *CTLA-4* mutated patients with granulomatous lymphocytic lung disease the use of Abatacept could lead to full or partial resolution in up to 70% of the treated patients [44^▪▪^]. As evidence of Abatacept use in *CTLA-4/LRBA* mutated patients is mainly derived from retrospective studies or case reports, a phase IIa prospective, nonrandomized, open-label, single arm multicenter trial (ABACHAI trial) is currently evaluating the safety and efficacy of Abatacept in patients with *CTLA-4* insufficiency or *LRBA* deficiency and results are expected to be published in 2023 [51^▪^].

Besides the promising results and the possibility of target treatments, HSCT still represents the only curative approach for both disorders; nevertheless, reported patients are scarce and with variable results [52,53]. Currently, recommendations on HSCT for these diseases are lacking and indications for HSCT are mainly directed towards those patients presenting multiple organs involvement refractory to conventional treatment and/or severe invasive infectious episodes [39,44^▪▪^,^52^–^54^]. As improving organ function is vital for achieving better HSCT outcomes, patients with CTLA-4 haploinsufficiency or LRBA deficiency might benefit from the use of Abatacept as a bridge to HSCT, rather than as continuative chronic treatment. In addition, the optimal minimal chimerism sufficient for achieving clinical and immunological control of the disease is still unclear [55^▪^]. Finally, the results of a CRISPR-Cas9 gene editing for *CTLA-4* haploinsufficiency have been recently published: the adopted approach led to CTLA-4 protein restoration and effective CD80/CD86 transendocytosis both in *in vitro* patients’ T cells and *in vivo ctla-4*^*−/−*^ mouse model, opening the possibility for a safer yet equally effective curative option than HSCT [56^▪▪^].

## CONCLUSION

In conclusion, CVID/CVID-like disorders present with heterogenous clinical and immunological manifestations, that may be caused by various genetic or environmental factors. In this review, we focalized our attention on three different monogenic forms of CVID-like disease and described new targeted strategies implicated in the different altered immunological pathways. The first one involves the PI3K pathway causing activated phosphoinositide 3-kinase delta syndrome, for which a direct oral inhibitor has been recently approved by the FDA. The other two involve T-regs maturation and homeostasis and are caused by defects in *CTLA-4* or *LRBA* gene; a soluble costimulation modulator (Abatacept) has been widely used showing good results in disease control. Targeted treatments in these disorders allow for significant control of noninfectious disease related complications, thus setting the basis for ameliorating prognosis and quality of life of affected patients. Furthermore, the costs and availability of these drugs may become a limitation depending on factors such as country of residence, type of national health system and others, especially since national and international guidelines for treatment of affected patients are not well defined yet. Understanding the biological mechanism of monogenic forms of CVID disorders may lead to the development of targeted therapies allowing for the application of precision medicine in the setting of IEIs, with evident implications for patients’ prognosis and quality of life.

## Acknowledgements

*We would like to thank the patient, the patient's family and the nurses for all their efforts. Several of us are members of the European Reference Network for Rare Immunodeficiency, Autoinflammatory and Autoimmune Diseases (project identification no. 739543).*

### Financial support and sponsorship

*The research leading to these results has received funding from the European Community's Seventh Framework Programme FP7/2007–2013 under grant agreement no 201549 (EURO-PADnet HEALTH-F2-2008-201549) and from the Italian Ministerial Grant GR-2010-2315762. The research leading to these results also received funding from the ‘Fondazione C. Golgi’, Brescia, Italy and the Jeffrey Modell Foundation.*

### Conflicts of interest

*There are no conflicts of interest.*

## REFERENCES AND RECOMMENDED READING

Papers of particular interest, published within the annual period of review, have been highlighted as:

▪ of special interest
▪▪ of outstanding interest
